# A comparison of quantitative methods for clinical imaging with hyperpolarized ^13^C‐pyruvate

**DOI:** 10.1002/nbm.3468

**Published:** 2016-01-18

**Authors:** Charlie J. Daniels, Mary A. McLean, Rolf F. Schulte, Fraser J. Robb, Andrew B. Gill, Nicholas McGlashan, Martin J. Graves, Markus Schwaiger, David J. Lomas, Kevin M. Brindle, Ferdia A. Gallagher

**Affiliations:** ^1^ Department of RadiologyUniversity of Cambridge, Addenbrooke's HospitalCambridgeUK; ^2^ Cancer Research UK Cambridge InstituteUniversity of Cambridge *Li Ka Shing Centre* CambridgeUK; ^3^ GE Global ResearchMunichGermany; ^4^ GE HealthcareClevelandOHUSA; ^5^ Nuclear Medicine, Klinikum rechts der IsarTechnical University MunichMunichGermany

**Keywords:** hyperpolarized carbon‐13, dynamic nuclear polarization, cancer imaging, spectroscopic imaging, kinetic modelling, quantitative analysis

## Abstract

Dissolution dynamic nuclear polarization (DNP) enables the metabolism of hyperpolarized ^13^C‐labelled molecules, such as the conversion of [1‐^13^C]pyruvate to [1‐^13^C]lactate, to be dynamically and non‐invasively imaged in tissue. Imaging of this exchange reaction in animal models has been shown to detect early treatment response and correlate with tumour grade. The first human DNP study has recently been completed, and, for widespread clinical translation, simple and reliable methods are necessary to accurately probe the reaction in patients. However, there is currently no consensus on the most appropriate method to quantify this exchange reaction. In this study, an *in vitro* system was used to compare several kinetic models, as well as simple model‐free methods. Experiments were performed using a clinical hyperpolarizer, a human 3 T MR system, and spectroscopic imaging sequences. The quantitative methods were compared *in vivo* by using subcutaneous breast tumours in rats to examine the effect of pyruvate inflow. The two‐way kinetic model was the most accurate method for characterizing the exchange reaction *in vitro*, and the incorporation of a Heaviside step inflow profile was best able to describe the *in vivo* data. The lactate time‐to‐peak and the lactate‐to‐pyruvate area under the curve ratio were simple model‐free approaches that accurately represented the full reaction, with the time‐to‐peak method performing indistinguishably from the best kinetic model. Finally, extracting data from a single pixel was a robust and reliable surrogate of the whole region of interest. This work has identified appropriate quantitative methods for future work in the analysis of human hyperpolarized ^13^C data. © 2016 The Authors. NMR in Biomedicine published by John Wiley & Sons Ltd.

**Abbreviations used**
DNP: dynamic nuclear polarization
SNR: signal‐to‐noise ratio
LDH: lactate dehydrogenase
NADH: reduced nicotinamide adenine dinucleotide
DCE: dynamic contrast‐enhanced
PET: positron emission tomography
SUV_*max*_: maximum standardized uptake value
AUC: area under the curve
EDTA: ethylenediaminetetraacetic acid
IDEAL: iterative decomposition with echo asymmetry and least‐squares estimation
CSI: chemical shift imaging
FID: free induction decay
FOV: field of view
ROI: region of interest
POI: pixel of interest
PIF: pyruvate inflow function
AICc: corrected Akaike information criterion
SD: standard deviation
TTP: time-to-peak
k_P_: forward exchange rate constant
k_L_: backward exchange rate constant
k_i_: pyruvate inflow rate
T_1eff_: effective T_1_
ρ: inverse of the effective T_1_
t_0_: inflow start time
t_e_: inflow end time

## Introduction

Functional and molecular imaging is increasingly used as a routine clinical tool in many areas of oncology. The advent of molecularly targeted drugs, combinational therapies, and personalized medicine has resulted in an increasing requirement for specific imaging methods to monitor drug efficacy; consequently, new imaging methods to probe tumour biology are required that demonstrate both intra‐ and inter‐patient repeatability. Dissolution dynamic nuclear polarization (DNP) is a new imaging test, which has the potential to image tissue biology. The technique increases the signal‐to‐noise ratio (SNR) of molecules containing one or more ^13^C nuclei by more than 10 000‐fold above thermal levels; this process is undertaken outside of the animal or patient, and the hyperpolarized molecule is subsequently injected intravenously 1. When combined with ^13^C‐MRSI, this increase in SNR allows real‐time *in vivo* metabolism of the molecules to be imaged non‐invasively 2.

There are now many ^13^C‐labelled probes that have been successfully hyperpolarized using DNP, and these have been used to interrogate many aspects of tissue biology and metabolism that occur in a wide range of disease processes 3, 4, 5, 6, 7, 8. [1‐^13^C]Pyruvate is the most extensively studied of these probes; the major reaction of pyruvate is its conversion into [1‐^13^C]lactate, which is catalysed by the enzyme lactate dehydrogenase (LDH) and requires the reduced form of nicotinamide adenine dinucleotide (NADH) as a cofactor. Hyperpolarized [1‐^13^C]pyruvate may also form [1‐^13^C]alanine, [1‐^13^C]bicarbonate, and [1‐^13^C]pyruvate‐hydrate in a pH‐dependent reaction 9. These reactions have been well characterized *in vivo* in pre‐clinical animal models of disease 10, 11, 12, 13, 14 and there are now a number of sites worldwide that are developing the technique for human use 15.

The success of hyperpolarized ^13^C‐labelled pyruvate as a cancer imaging biomarker is largely dependent on the phenomenon of aerobic glycolysis within tumours known as the Warburg effect 16. There is a high lactate concentration within most cancers, even in normoxic conditions, and the hyperpolarized ^13^C signal may rapidly exchange between the injected [1‐^13^C]pyruvate and the endogenous lactate pool. Imaging of this exchange reaction in animal models has been shown to detect early treatment response 17, 18 and correlate with tumour grade 19. The results from the only clinical study performed to date have shown that labelled lactate may be present in small tumours that are not visible with standard proton imaging techniques 15.

The sensitivity of ^13^C‐MRSI to small changes in metabolic rate offers the potential to non‐invasively monitor metabolic alterations in patients. However, for any novel imaging technique to be widely adopted, it must be repeatable and reproducible, and will ideally utilize simple and robust quantitative methods for analysis. Current clinical approaches for the analysis of dynamic contrast‐enhanced (DCE) MRI and positron emission tomography (PET) can offer insight into quantitative imaging with DNP. For example, the maximum standardized uptake value (SUV_max_) is a very simple and powerful routine clinical tool to quantify metabolism in PET imaging with ^18^F‐fluorodeoxyglucose 20. For each voxel of a PET image, the SUV is defined as the tissue radioactivity concentration divided by the injected radiation per unit body weight, correcting for decay from the time of injection. The SUV_max_ is the voxel of highest SUV within a region of interest (ROI), while the SUV_mean_ is the average over that region*.* A number of methods have been employed to quantify the pyruvate–lactate exchange reaction measured using DNP, many of which involve fitting kinetic models of varying complexity to imaging or spectroscopy data to produce the forward reaction rate constant *k*_P_ as a quantitative marker 17, 19, 21, 22. Simple methods for estimating *k*_P_ from the time course of pyruvate and lactate have been suggested 23, as well as methods using model‐free parameters such as the area under the metabolite curve (AUC) 24, or ratios of the metabolite signal peaks 25, 26. However, there is currently no consensus on the best method to characterize the pyruvate–lactate reaction, either for research purposes or for more routine clinical use.

In this study, we used both *in vitro* and *in vivo* dynamic hyperpolarized data to comprehensively compare a range of kinetic models and simple analysis parameters. We used a clinical hyperpolarizer and imaging sequences with a 3 T MR system, as well as a pyruvate concentration similar to the blood pyruvate concentration we anticipate in future patient studies. The aim was to determine which quantitative parameters are most appropriate to describe the dynamic time‐course data acquired, testing for accuracy, simplicity and robustness in each case. Each analysis was also applied *in vivo* to rats with subcutaneous tumours, which were imaged with the same spectroscopic imaging sequence to examine the effect of pyruvate inflow.

## Materials and Methods

### *In vitro* experiments

Research grade fluid paths (GE Healthcare, Milwaukee, WI, USA) were filled with 100 μl of [1‐^13^C]pyruvic acid doped with 15 mM of an electron paramagnetic agent (trityl radical AH111501, GE Healthcare, Milwaukee, WI, USA) and 30 ml of dissolution fluid containing 1 g l^−1^ ethylenediaminetetraacetic acid (EDTA, GE Healthcare). Samples were polarized using a clinical hyperpolarizer (SPINlab, GE Healthcare) at approximately 0.9 K and 5 T for an average of 101 min (range 83–149 min) to an average polarization of 21% (range 10–38%) at the time of measurement using a benchtop NMR polarimeter (Oxford Instruments, Abingdon, UK). Following rapid dissolution, 1.4 ml of neutralization medium containing 0.72 M NaOH, 0.4 M Tris buffer and 0.1 g l^−1^ EDTA (GE Healthcare) was added to the solution. The final pH ranged from 6.7 to 7.4 with an average of pH 7.2.

Imaging phantoms consisted of 15 ml Falcon tubes filled to 14 ml with fivefold concentrated phosphate buffered saline at pH 7.2 and containing the coenzyme NADH at 4.4 mM (Sigma‐Aldrich, Gillingham, UK). L‐LDH from rabbit muscle (Sigma‐Aldrich, Gillingham, UK) was added in quantities varying from 0 to 120 U, a range chosen to incorporate the expected range of *k*_P_ in human blood based on the only published study to date (mean ± standard deviation (SD) of 0.045 ± 0.025 s^−1^) 15. 1 ml of the above 60 mM hyperpolarized solution was added simultaneously to the three tubes making up each set immediately before imaging, giving a final pyruvate concentration of about 4 mM. This is similar to the final blood concentration in the rats (~3 mM) and that expected in patients (~1.5 mM). If uniform distribution is achieved in the body in the timescale of the half‐life of hyperpolarization, then the tissue concentrations would be lower. Neither pyruvate nor NADH were rate limiting. The time between dissolution and imaging was approximately 35 s.

### *In vivo* experiments

Four adult female Fischer 344 rats (Charles River, Sulzfeld, Germany; 165 ± 6 g body weight) bearing subcutaneous mammary adenocarcinomas were imaged 27, 28. Tumours were induced by implanting 1 × 10^6^ MAT B III cells (syngenic breast cancer cell line), and imaging was performed 12–16 days after cell implantation. Animals were anesthetized with 1–3% isoflurane, monitored for ECG, breathing, and temperature, and kept warm on a heating pad with circulating warm water. The time delay between dissolution and injection was 15–20 s. The animal study was approved by the local governmental committee for animal protection and welfare (Tierschutzbehörde, Regierung von Oberbayern).

[1‐^13^C]Pyruvic acid doped with 15 mM of the trityl radical OX063 and 1 mM gadoteric acid (Guerbet, Paris, France) was polarized in a HyperSense DNP polarizer (Oxford Instruments) for approximately 45 min at 1.4 K and 3.35 T to a polarization of approximately 25%. Dissolution fluid, containing 80 mM NaOH, 80 mM Tris buffer, and 0.1 g l^−1^ EDTA dissolved in water, was heated to 185 °C and used to rapidly dissolve the polarized sample. The final solution contained 80 mM [1‐^13^C]pyruvate at pH 7.6 and physiological temperature and osmolarity. This was injected into a tail vein inside the MRI scanner at a rate of approximately 0.2 ml s^−1^ and at a dose of 2.5 ml kg^−1^. Dynamic imaging was performed from the time of injection.

### Spectroscopic imaging

All imaging was carried out on a clinical 3 T MRI system (Signa HDx, GE Healthcare) using imaging sequences from the Multinuclear Spectroscopy (MNS) research pack Version 2.0 (GE Global Research, Munich, Germany). Enzyme phantoms were imaged in sets of three using a ^13^C–^1^H multi‐nuclear receive/transmit coil (GE Coils, Aurora, OH, USA). First, four 90° non‐localized spectral–spatial pulses with *T*_R_ = 1 s centred on the lactate Larmor frequency were applied 27; this was found to be the optimum number for removing signal from any lactate labelling prior to commencement of the experiment whilst retaining maximum pyruvate polarization. These were immediately followed by a 3 min IDEAL (iterative decomposition with echo asymmetry and least‐squares estimation) spiral chemical shift imaging (CSI) acquisition 29 acquired using a single 20 mm axial slice. Each excitation is followed by a single‐shot spiral image encoding module, with echo‐time shifting of 1.12 ms between excitations. Seven time‐shifted echoes plus a single free induction decay (FID) spectrum are acquired in total for each time step, with the chemical‐shift information from the FID providing prior knowledge for the reconstruction. Other parameters were *T*_R_ 500 ms, flip angle 5°, field of view (FOV) 80 mm, nominal matrix resolution 32 × 32 and 4 s time resolution.

Animals were imaged on a similar clinical 3 T MRI system (Signa HDx, GE Healthcare) using a rat‐sized ^13^C–^1^H multi‐nuclear birdcage coil. The same IDEAL spiral CSI acquisition was performed through four axial 10 mm slices over 1 min with a temporal resolution of 4 s. ^13^C‐pyruvate is replenished by inflowing blood *in vivo*, so a larger flip angle of 10° was used. All other parameters were consistent with the *in vitro* experiments. Single metabolite *k*‐space data was first reconstructed by matrix inversion with off‐resonance correction, followed by Cartesian regridding for spatial reconstruction 29. Gaussian *k*‐space filtering was applied during post‐processing on both data sets, resulting in an effective image resolution of 5 × 5 mm^2^. For anatomical reference, standard gradient echo proton images were acquired from the same slice geometry and FOV (resolution 256 × 256, slice thickness 3 mm, spacing 7 mm, *T*_E_ 10 ms, *T*_R_ 500 ms).

### Data analysis

Imaging data was exported into MATLAB (MathWorks, Natick, MA, USA) for analysis using custom built software. Images were partially noise‐corrected by subtracting the average squared background noise from the power images 30. Dynamic time‐course data for pyruvate and lactate were extracted from the images, using two methods for comparison. In the first, an ROI was defined by thresholding the *t* = 0 pyruvate image at 40% of the maximum pyruvate signal within each phantom for the *in vitro* data, or by outlining the tumour boundary based on the anatomical proton image for the *in vivo* data. This ROI was applied to all subsequent images in the series and the pixels within the ROI were averaged to produce the relative signal strengths of each metabolite at each time point. The second method defined a pixel of interest (POI) as the pixel within each phantom, or within the rat tumour, that demonstrated the highest lactate signal after averaging over all time points. Dynamic data from these two extraction methods was separately analysed using each of the methods detailed below.

### Kinetic modelling

The exchange reaction between pyruvate and lactate, along with the irreversible hyperpolarized signal loss due to spin–lattice relaxation and applied RF excitation, can be characterized by the following coupled differential equations:

$$\begin{array}{l} \frac{dP\left(t\right)}{dt}=k_{L}L\left(t\right)-k_{P}P\left(t\right)-\mathrm{\rho P}\left(t\right)\\ \frac{dL\left(t\right)}{dt}=k_{P}P\left(t\right)-k_{L}L\left(t\right)-\mathrm{\rho L}\left(t\right) \end{array}$$

where *P* and *L* are the relative pyruvate and lactate signal intensities, *k*_P_ and *k*_L_ are the forward and backward reaction rate constants respectively and *ρ* is the effective relaxivity given by

$$\rho =\frac{1}{T_{1\;\mathrm{eff}}}=\frac{1}{T_{1}}-\frac{1}{t_{R}}\ln\left(\cos\theta \right)$$

where *T*_1_ is the relaxation time of pyruvate in the medium or tissue, *t*_R_ is the known repetition time of the applied RF pulses and *θ* is the flip angle. To reduce the number of parameters to be fitted, we assume throughout that the pyruvate and lactate relaxivities are equal, which in the limit of fast exchange between metabolites can be shown to be a good approximation 31, and use the above expression to correct for RF flip angle. Each model variant was applied to yield the forward reaction rate constant *k*_P_, the *T*_1_ decay constant, their standard errors and other fitting parameters where appropriate. Constrained fits were carried out in MATLAB using the *fmincon* function unless otherwise stated. Solutions were found by minimizing the negative logarithm of the maximum likelihood function, which for *N* data points is given by

$$f\left(K,\sigma \right)=\sum_{i=1}^{N}\frac{{\left(L_{i}-L_{i}^{\text{data}}\right)}^{2}}{2\sigma^{2}}-\ln\left(1-\exp\left\{-\frac{{\left(P_{i}-P_{i}^{\text{data}}\right)}^{2}}{2\sigma^{2}}\right\}\right)+\ln\left({\left(P_{i}-P_{i}^{\text{data}}\right)}^{2}\right)$$

Here ***K*** is the vector of free parameters to be fitted, *σ* ^2^ is the lactate variance, *L*_*i*_ and *P*_*i*_ are the model estimates of the metabolite signal strengths and *L*_*i*_^data^ and *P*_*i*_^data^ the measured values for the *i*th data point. This likelihood function is derived from a Bayesian extension to least squares minimization that addresses the disproportionately large early pyruvate signal, as described by Hill *et al* 24. It incorporates a lower‐bound prior on the pyruvate noise, which acts to reduce the impact of any large early pyruvate residuals which can otherwise dominate the fit. Due to the sensitivity of the optimization algorithm to the initial values of *P* and *L*, we used a Monte Carlo method to randomly vary these inputs over 1000 runs for each model fit. Inputs were allowed to vary over a Gaussian distribution centred, with an SD of 5%, on the measured value of *P* or *L* at *t* = 0.

#### The two‐site exchange model

For the *two‐way differential* model, the above set of differential equations were solved simultaneously to fit the pyruvate and lactate time‐course data with *k*_P_, *k*_L_ and *ρ* as free parameters. The equations may also be solved analytically to give the following solutions:

$$\begin{array}{l} P\left(t\right)=\frac{1}{k_{P}+k_{L}}\left\{P\left(0\right)\left(k_{L}e^{-\mathrm{\rho t}}+k_{P}e^{-\left(k_{P}+k_{L}+\rho \right)t}\right)+L\left(0\right)\left(k_{L}e^{-\mathrm{\rho t}}-k_{L}e^{-\left(k_{P}+k_{L}+\rho \right)t}\right)\right\}\\ L\left(t\right)=\frac{1}{k_{P}+k_{L}}\left\{P\left(0\right)\left(k_{P}e^{-\mathrm{\rho t}}-k_{P}e^{-\left(k_{P}+k_{L}+\rho \right)t}\right)+L\left(0\right)\left(k_{P}e^{-\mathrm{\rho t}}+k_{L}e^{-\left(k_{P}+k_{L}+\rho \right)t}\right)\right\} \end{array}$$

These equations themselves may then be fitted directly to the data in the *two‐way integral* model, which is computationally faster than applying the full differential fit but may be more sensitive to the initial values of the parameters. For the *in vitro* data, the latter two models are virtually identical apart from the fitting algorithms used; however, they require different approximations for metabolite inflow when used *in vivo.* A popular simplification of the model is to set the backward reaction rate constant *k*_L_ to zero 19, 21, 22, 32, 33. This is argued to be a reasonable assumption, since *k*_L_ is often around 1/10 of the forward rate constant *k*_P_ and, depending on the model used, may not be a mathematically distinct parameter. To investigate the validity of the *one‐way model*, the above integral solutions with *k*_L_ = 0 were also applied to the data.

#### Ratiometric model

A *ratiometric model* has been suggested by Li *et al* 31. Briefly, when the ratio is taken of the above integral solutions, *ρ* can be eliminated as a parameter; this new model can be fitted to the ratio of the lactate‐to‐pyruvate data to solve for the rate constants *k*_P_ and *k*_L_. Prior to fitting with the *nlinfit* function in MATLAB, ratio data was smoothed by averaging over every three data points. The lactate data was used to weight the fit towards the region in which the rate constants are stable and *ρ* was subsequently derived as a single unknown by inputting the *k*_P_ and *k*_L_ obtained from the ratiometric fit into the differential kinetic model.

### Model‐free methods

#### Fall minus rise at height α approach

Pagès and Kuchel suggest a method for estimating *k*_P_ and *ρ* from graphical features of the time course, which they call the *FmR*_*α*_ (fall minus rise at height *α*) approach 23. They suggest parameter *β* to be the ratio of the lactate‐to‐pyruvate signals for the time at which the lactate signal is maximal, then show this to be mathematically equal to the product of *k*_P_ and *T*_1 eff_. Furthermore, they show that an estimate for *T*_1 eff_ can be obtained from the width of the lactate curve at a specific height *α*. Although they suggest it is sufficient to use a consistent value of *α* = 0.8, we calculated *α* explicitly in each case to avoid incurring a non‐physical correlation between *k*_P_ and *T*_1_.

#### Lactate–pyruvate peak ratio

The *in vitro T*_1 eff_ is only affected by physical factors such as magnetic field and temperature. Since these factors are controlled for, *T*_1 eff_ is not expected to show a large variation. The above parameter *β*, which we re‐name the *L*–*P peak ratio*, could therefore be expected to correlate with LDH concentration, and as such was investigated as a potential parameter of interest.

#### AUC ratio

The ratio of the lactate‐to‐pyruvate AUC has been shown by Hill *et al.* to be independent of the shape of the pyruvate inflow 24, making it an excellent candidate for analysis of *in vivo* data. If it is assumed that only the pyruvate has an explicit input function and that the concentration of both metabolites is zero at *t* = 0, then using the two‐way, two‐site model the AUC ratio can be shown to be proportional to *k*_P_. For *in vitro* data this ratio was calculated in two ways: first taking the AUC ratio of the available data (*AUC data*) and second fitting bi‐exponential solutions extrapolated back to *t* = 0 (*AUC fit*).

#### Time‐to‐peak measurements

Finally, we suggest a new approach that examines the time difference from the start of the lactate build up to its peak, as has been used in DCE‐MRI. Starting from the two‐way model, the rate equations can be solved analytically to find the time at which the lactate reaches its maximum by differentiating the lactate solution, and solving for *t* when this is zero*.* The time‐to‐peak (TTP) is then given by

$$\mathrm{TTP}=\frac{1}{k_{P}+k_{L}}.ln\left\{1+T_{1\;\mathrm{eff}}\left(k_{P}+k_{P}\right)\right\}$$

**Figure 1:**
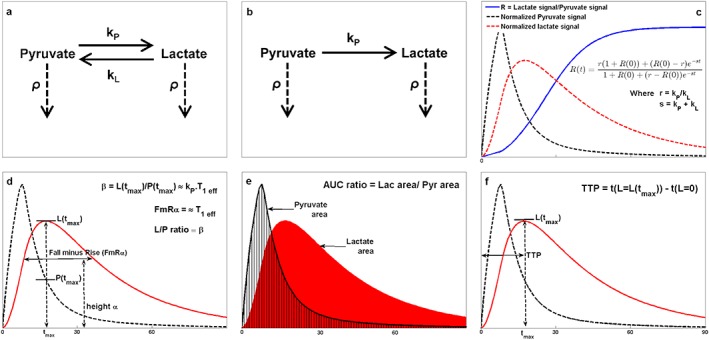
Schematic diagrams for each of the models and model‐free methods proposed. (a) Interactions accounted for in the two‐way differential/integral models where *k*_P_ and *k*_L_ are the forward and backward reaction rate constants respectively and *ρ* is the inverse of the effective spin–lattice relaxation, *T*_1 eff_. (b) Interactions for one‐way integral model. (c) Example metabolite time courses demonstrating the ratiometric model. (d) *FmR*_*α*_ approach and L–P ratio method. (e) Lactate‐to‐pyruvate AUC ratio. (f) Lactate TTP.

The TTP should show a roughly inverse correlation with LDH concentration. A bi‐exponential function was fitted to the time‐domain lactate data and extrapolated back to find the predicted start time of the increase in lactate signal from zero. Schematic diagrams of each of the models and model‐free methods described above are presented in Figure 1a–f.

#### *In vivo* imaging

*In vivo* analysis of pyruvate metabolism is complicated by the flow of injected metabolites in the bloodstream, tissue diffusion and cellular transport. For full modelling of this data, a pyruvate inflow function (PIF) must be included to describe the large initial pyruvate peak as the injected bolus reaches the tissue of interest. In the interest of simplicity, each of the four model variants was fitted only to data from the pyruvate peak onwards, which in each case occurred at the third data point. Each of the model‐free methods was implemented on the entire dataset. A common way to approximate the PIF is to use a box‐car or trapezoidal function, and an alternative is to fit a piecewise integral solution. The latter involves splitting the pyruvate profile into two segments described by separate equations: the first for constant pyruvate inflow, and the second describing signal decay and conversion to lactate 19, 34, 35, 36. Here we have used a Heaviside step function to incorporate a continuous PIF into the pyruvate two‐way differential equation with the same characteristics as a box‐car function:

$\frac{dP\left(t\right)}{dt}=k_{i}\left(1-\frac{1}{1+e^{-2\left(t-t_{e}\right)}}\right)+k_{L}L\left(t\right)-k_{P}P\left(t\right)-\mathrm{\rho P}\left(t\right)$ where *k*_i_ is the pyruvate flow rate and *t*_e_ is the time at which the inflow ceases. We compared this to the *piecewise one‐way integral model* described by Zierhut *et al.* 19 in two ways: fixing *t*_e_ at the peak of the pyruvate curve or fitting for it as an extra parameter.

### Statistical analysis

The quantitative parameters derived using each method were tested for their correlation with the known *in vitro* LDH concentration. These correlations were generally linear. Both Pearson (linear) and Spearman (rank) correlation coefficients and their two‐tailed *p*‐values were calculated, so that it was possible to compare all methods directly whilst making no assumptions about the nature of the dependences. In order to determine whether one analysis approach was significantly better than another, Steiger's *z*‐test for dependent correlations was implemented pairwise on the Pearson then Spearman coefficients produced by each method, and the two‐tailed *p*‐values calculated (significance *p* < 0.05) 37. In addition, a simple linear regression model was applied in each case to calculate the adjusted *R* ^2^ values both with and without a robust bi‐square weighting function applied to the data. The difference in *R* ^2^ with and without the weighting, denoted the ‘instability’ factor, provides a crude indicator of how much the fit is dominated by outliers and therefore how robust a particular method may be. The mean, range and SD of *T*_1_ values obtained from all phantoms taken together were also analysed for each quantitative method to assess goodness of fit. Since *T*_1_ is expected to be highly consistent *in vitro*, a large range and high SD suggests that a model is not fully describing the data or is underparameterized.

To assess how well each model was able to fit the *in vivo* data, the corrected Akaike information criteria (AICc) were calculated 38. The AICc uses the minimized likelihood function values to examine how well each model is able to describe the data, whilst imposing a penalty for each extra free parameter to guard against overfitting; a low AICc denotes higher model accuracy. The relative likelihood that each model is correct as compared to the model with the lowest AICc was then calculated. To investigate model‐free parameters *in vivo*, correlation coefficients and *R* ^2^ values were again calculated; however because the enzyme activity was an unknown, they were compared against *k*_P_ values derived using the most likely model with ROI or POI data as appropriate. The ROI and POI results were combined when calculating correlation coefficients due to the small size of the dataset.

## Results

### *In vitro* modelling

**Figure 2:**
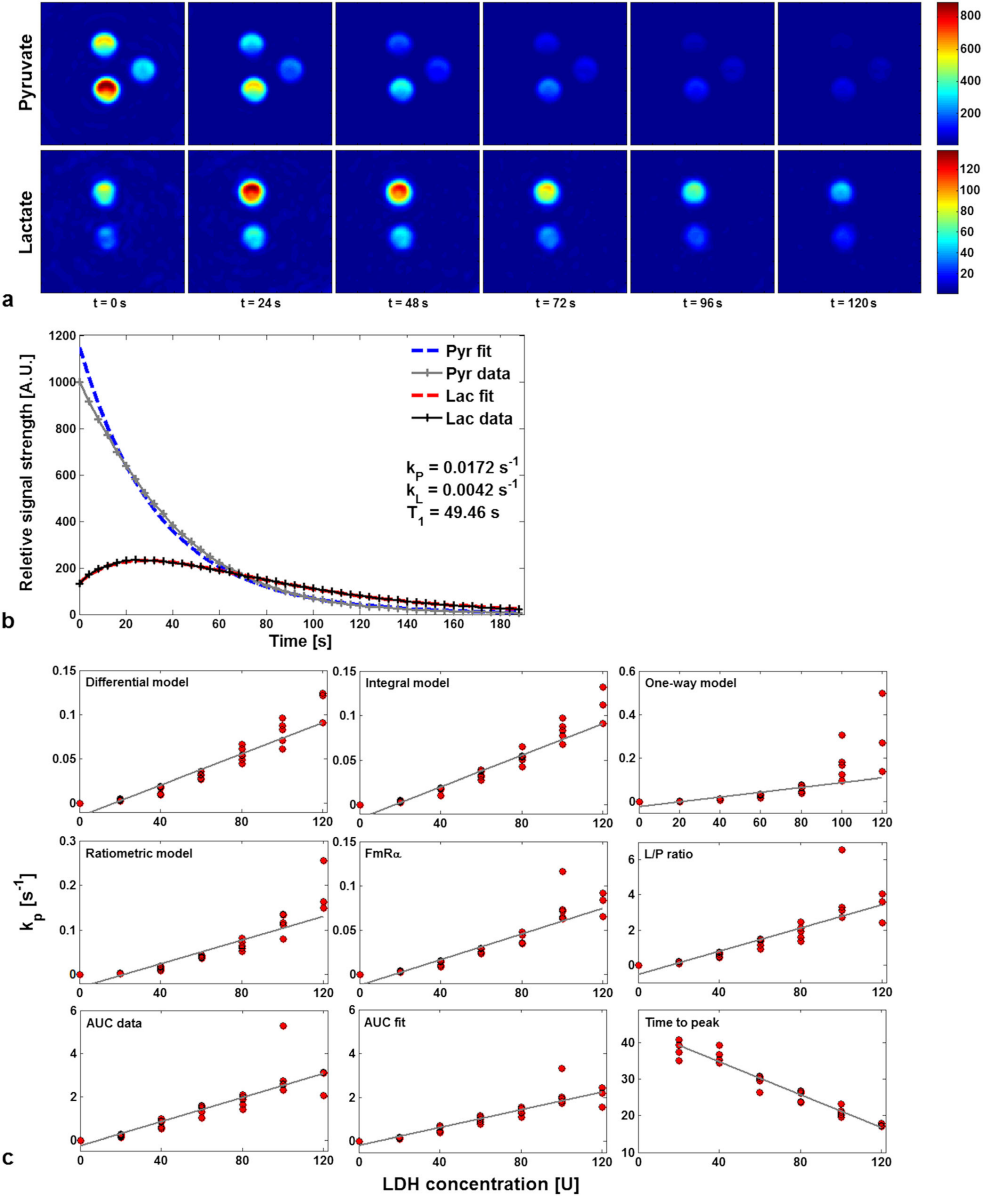
Representative data from the *in vitro* study. (a) IDEAL spiral CSI axial images of pyruvate and lactate in three phantoms, displayed at 24 s intervals. Phantoms contain 40, 0 and 20 U of LDH enzyme (clockwise from top); the colour bar shows arbitrary signal units scaled to the brightest point in the time curve for each metabolite. (b) Pyruvate and lactate time courses (solid lines) extracted from the 40 U phantom using the ROI method and fitted using the differential two‐way kinetic model (dashed lines). (c) Correlation with LDH enzyme concentration of the forward reaction rate constants *k*_P_ derived from four model variants (differential, integral, ratiometric and one‐way models). Note that the scale for *k*_P_ varies between plots.

Thirty‐three 15 ml phantoms containing 0 U (*n* = 2), 20 U (*n* = 5), 40 U (*n* = 6), 60 U (*n* = 6), 80 U (*n* = 6), 100 U (*n* = 5) and 120 U (*n* = 3) of LDH enzyme were imaged in sets of three, for which representative images can be seen in Figure 2a. Ringing artefacts, noise spikes and other interference were visible in some images; however, since it was of particular interest to test the quantitative analysis methods for robustness against such common artefacts, none of the data was excluded. The quantitative parameters obtained with each analysis method were tested for their correlation with the known scale of phantom LDH concentrations. The two‐tailed *p*‐values calculated from the Pearson and Spearman correlation coefficients were all highly significant (*p* < 0.001).

The results of the analyses are summarized in Table 1. For the model‐based approaches, application of Steiger's *z*‐test to the Pearson coefficients for the ROI data demonstrated the ratiometric method to be significantly poorer than the other three models, which were not found to be significantly different. However, the POI data showed the one‐way model to be worse than the other methods tested. In both cases, the differential and integral models are better able to constrain *T*_1_ compared to the other three methods, which suggests that they are more closely modelling the data. A fit with the differential model is shown in Figure 2b. The variation in *k*_P_ between the integral and differential versions of the two‐way model is a function of the algorithm used for fitting the data, but this difference was shown to be insignificant (*p* > 0.2)*.* Despite the simplicity of the *FmR*_*α*_ approach, *k*_P_ values obtained from the *FmR*_*α*_ analysis were not significantly different from those obtained by modelling methods, other than the ROI integral model.

**Table 1:** Summary of the analysis for the *in vitro* data are shown by comparing the calculated exchange rate constants with the known enzyme concentration. Calculations have been performed using both the ROI and POI approaches

ROI	Pearson	Spearman	Adj. *R* ^2^	Adj. *R* ^2^ robust	Instability	*T*_1_ mean	*T*_1_ range	*T*_1_ SD
*k*_P_ differential	0.950	0.984	0.900	0.917	0.017	52.4	13.2	3.04
*k*_P_ integral	0.952	0.986	0.903	0.931	0.028	52.5	13.5	2.97
*k*_P_ one‐way	0.942	0.982	0.883	0.907	0.025	58.4	21.7	5.99
*k*_P_ ratiometric	0.896	0.986	0.796	0.891	0.095	57.3	26.9	5.46
*k*_P_ *FmR*_*α*_	0.920	0.981	0.842	0.923	0.082	47.7	21.9	4.36
L–P ratio	0.854	0.969	0.721	0.905	0.184	–	–	–
AUC data	0.877	0.966	0.761	0.945	0.184	–	–	–
AUC fit	0.916	0.974	0.833	0.944	0.110	–	–	–
TTP	−0.971	−0.964	0.940	0.946	0.006	–	–	–
POI	Pearson	Spearman	Adj. *R* ^2^	Adj. *R* ^2^ robust	Instability	*T*_1_ mean	*T*_1_ range	*T*_1_ SD
*k*_P_ differential	0.925	0.982	0.852	0.946	0.094	53.8	12.4	3.75
*k*_P_ integral	0.919	0.982	0.840	0.939	0.100	54.0	15.8	4.23
*k*_P_ one‐way	0.767	0.924	0.574	0.923	0.348	60.2	26.6	7.78
*k*_P_ ratiometric	0.915	0.984	0.831	0.852	0.020	58.6	30.4	6.63
*k*_P_ *FmR*_*α*_	0.922	0.981	0.846	0.942	0.096	46.4	21.8	4.81
L–P ratio	0.858	0.976	0.727	0.921	0.195	–	–	–
AUC data	0.880	0.971	0.766	0.926	0.160	–	–	–
AUC fit	0.898	0.976	0.801	0.943	0.142	–	–	–
TTP	−0.963	−0.964	0.926	0.921	0.005	–	–	–

In contrast, the model‐free methods showed greater variation, with the TTP proving to be significantly better, and the L–P ratio significantly worse, than other methods tested. The AUC from the extrapolated fits also performed well, being indistinguishable from the modelling approaches. When *p*‐values were calculated from the Spearman coefficients, the results largely supported those from the Pearson coefficients with the exception of the TTP, which performed least well. Table 2 shows full significance matrices for both ROI and POI data from the pairwise application of Steiger's *z*‐test.

**Table 2:** Significance matrix for *in vitro* data from ROI data (top) and POI data (bottom). Matrix shows *p*‐values from applying Steiger's *z*‐test for comparing the relative strength of correlations pairwise to Pearson coefficients; the Pearson coefficients test the correlation between quantitative parameters produced by each method, and known enzyme concentration. * indicates that the better ranking (high 1–9 low) analysis method of the two is significantly so (significance level *p* < 0.05)

ROI analysis	Pearson	Raw rank	*k*_P_ integral	*k*_P_ one‐way	*k*_P_ ratiometric	*k*_P_ *FmR*_*α*_	L–P ratio	AUC data	AUC fit	TTP
*k*_P_ differential	0.9503	3	0.762	0.664	0.001*	0.075	0.001*	0.007*	0.098	0.156
*k*_P_ integral	0.9518	2	–	0.604	0.000*	0.050*	0.000*	0.005*	0.075	0.183
*k*_P_ one‐way	0.9415	4		–	0.032*	0.402	0.011*	0.051	0.336	0.219
*k*_P_ ratiometric	0.8956	7			–	0.380	0.322	0.643	0.539	0.001*
*k*_P_ *FmR*_*α*_	0.9201	5				–	0.000*	0.010*	0.722	0.010*
L–P ratio	0.8541	9					–	0.078	0.000*	0.000*
AUC data	0.8766	8						–	0.000*	0.000*
AUC fit	0.9158	6							–	0.007*
TTP	−0.9705	1								–
POI analysis										
*k*_P_ differential	0.9254	2	0.206	0.000*	0.640	0.753	0.003*	0.073	0.173	0.054
*k*_P_ integral	0.919	4	–	0.000*	0.860	0.763	0.007*	0.129	0.303	0.035*
*k*_P_ one‐way	0.7666	9		–	0.002*	0.000*	0.048*	0.026*	0.003*	0.000*
*k*_P_ ratiometric	0.9147	5			–	0.780	0.173	0.375	0.647	0.020*
*k*_P_ *FmR*_*α*_	0.9223	3				–	0.000*	0.024*	0.103	0.048*
L–P ratio	0.8575	8					–	0.103	0.001*	0.001*
AUC data	0.8795	7						–	0.074	0.003*
AUC fit	0.8983	6							–	0.010*
TTP	−0.9633	1								–

The sensitivity of *k*_P_ may be examined in terms of the offsets of the fit lines at the origin. The cause of these offsets is twofold: first there is a positive skew caused by the divergent *k*_P_ values produced at high LDH concentrations, and second sensitivity limitations at very low concentrations cause an initial monotonic but non‐linear rise in *k*_P_ with increasing LDH. The relative sensitivity of each model to these features is characterized by the difference in the two correlation coefficients; they are accounted for well by the Spearman coefficients, which allow regions of monotonic non‐linear increase, but will incur a penalty with the Pearson coefficients, which enforce linearity.

### *In vivo* modelling

Each of the four models tested *in vitro* were fitted to both ROI and POI derived time‐course data from four rats with subcutaneously implanted tumours. An additional three models were tested which incorporated a PIF. The AICc scores and the relative likelihood of each model correctly describing the observed data were then calculated; the results of this, along with the *T*_1_ mean, range and SD, are shown in Table 3. The *T*_1_ values obtained were slightly higher than those previously estimated in healthy rats of about 15 s, but consistent with other rodent tumour *T*_1_ values 19, 39. The *in vivo* results were similar to those found *in vitro*, with the differential and integral models statistically indistinguishable from each other, but significantly more likely than the one‐way or ratiometric models to accurately describe the data. Models incorporating a PIF were applied to a larger number of data points than the remaining models to include data prior to the pyruvate peak. For this reason, the relative likelihood values for the PIF models were calculated separately to compare just these three, as shown in Table 3.

**Table 3:** Summary of statistical analysis from calculating the AICc for each model, separately fitted to ROI and POI data from four rats with subcutaneously implanted tumours. The relative likelihoods for models incorporating a PIF were calculated separately against each other. The lowest section shows correlation coefficients for model‐free parameters against *k*_P_ values from the differential Heaviside step PIF model. * *p* < 0.05; ** *p* < 0.001

ROI	Average AICc	Relative likelihood	*T*_1_ mean	*T*_1_ range	*T*_1_ SD
*k*_P_ differential	368.9	0.257	25.7	7.51	2.78
*k*_P_ integral	367.5	1	25.4	7.60	2.90
*k*_P_ one‐way	403.3	2.95 × 10^−16^	23.2	5.98	2.26
*k*_P_ ratiometric	388.8	5.87 × 10^−10^	27.1	8.54	3.03
*k*_P_ differential, PIF	469.0	1	24.6	6.90	2.61
*k*_P_ piecewise, fixed *t*_e_ PIF	495.9	2.12 × 10^−12^	22.2	5.71	2.24
*k*_P_ piecewise, variable *t*_e_ PIF	499.2	2.12 × 10^−12^	22.2	5.78	2.28
POI
*k*_P_ differential	436.1	1	25.0	4.75	1.83
*k*_P_ integral	436.2	0.880	25.2	4.84	1.89
*k*_P_ one‐way	449.5	1.51 × 10^−6^	22.8	5.18	1.97
*k*_P_ ratiometric	469.0	5.28 × 10^−15^	27.6	2.47	0.91
*k*_P_ differential, PIF	527.8	1	23.9	4.59	1.73
*k*_P_ piecewise, fixed *t*_e_ PIF	533.7	2.97 × 10^−3^	22.5	5.82	2.13
*k*_P_ piecewise, variable *t*_e_ PIF	536.4	1.90 × 10^−4^	22.0	5.53	2.15
Combined ROI and POI	Pearson	Spearman	Adj. *R* ^2^	Adj. *R* ^2^ robust	
*k*_P_ *FmR*_*α*_	0.797*	0.714	0.574	0.497	
L–P ratio	0.636	0.548	0.305	0.637	
AUC	0.888*	0.905*	0.754	0.733	
TTP	−0.970**	−1.00**	0.930	0.920	

**Figure 3:**
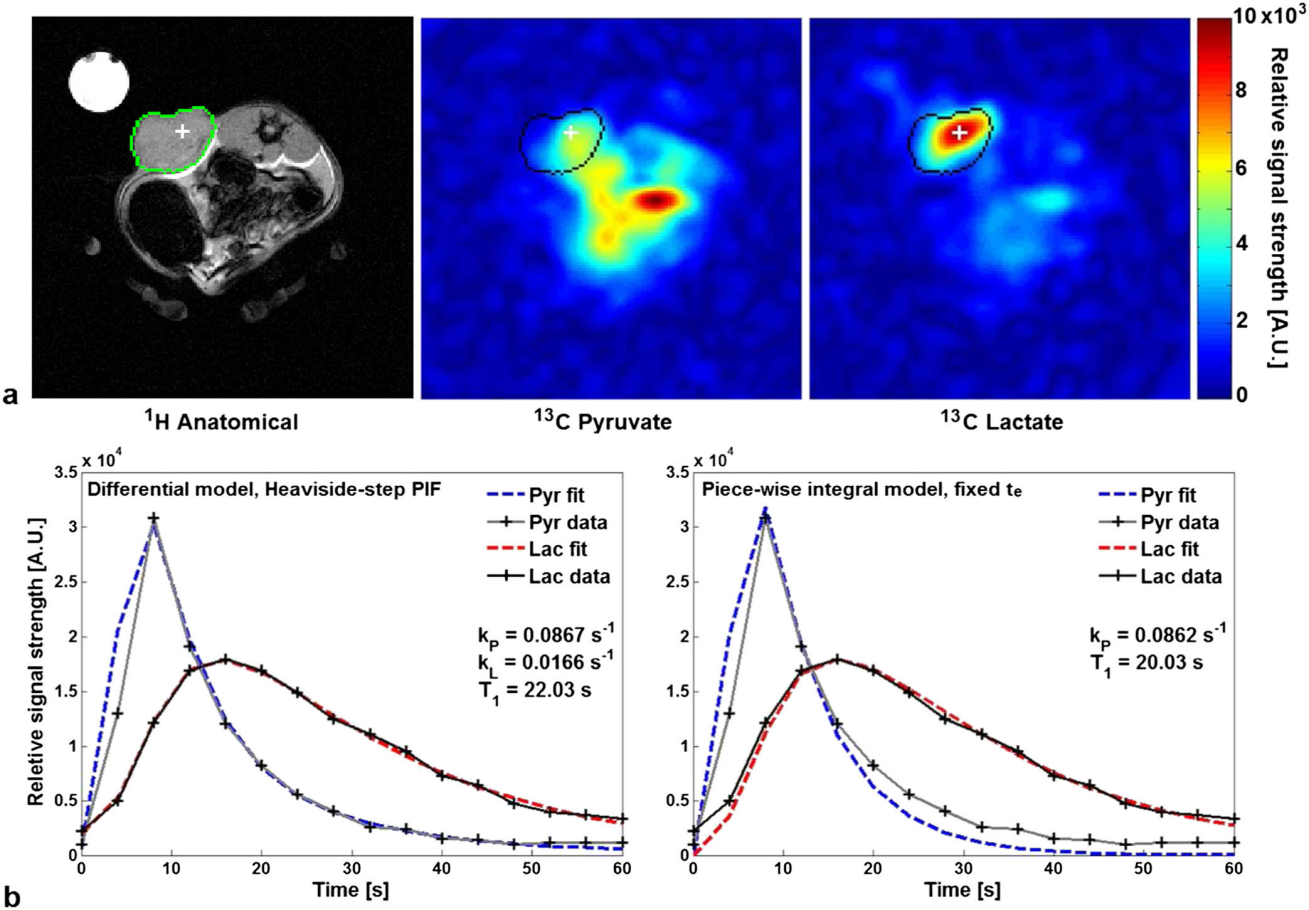
*In vivo* data from a rat (Rat 1) with a subcutaneous implanted mammary adenocarcinoma. (a) Proton anatomical reference image and hyperpolarized ^13^C‐pyruvate and ^13^C‐lactate images at *t* = 20 s; colour bar in arbitrary signal units. (b) Pyruvate and lactate time courses (solid lines) extracted from the thresholded tumour ROI and fits (dashed lines) from the differential kinetic model with a Heaviside step PIF (left) and the fixed *t*_e_ piecewise model (right) for comparison.

The differential model with the Heaviside step PIF provided the lowest AICc of the three, fitting the data significantly better than the piecewise models. Interestingly, the penalty for allowing the end of inflow time *t*_e_ to vary as an extra free parameter within the piecewise model was greater than the improvement this made to the fit. It has been stated by previous users of the model that *t*_e_ should correspond to the injection length starting from *t*_0_ 19, 35, which in the case of the rats was around 2 s. The fitted *t*_e_ calculated here was 8–10 s, which corresponded to the pyruvate peak time instead. For this reason, *t*_e_ was then fixed at the pyruvate peak time, as was the centre of the downslope for the Heaviside step function used in the differential model. Figure 3b shows fits of the differential PIF model as compared with the fixed *t*_e_ piecewise model to ROI time courses from one animal (Rat 1).

**Figure 4:**
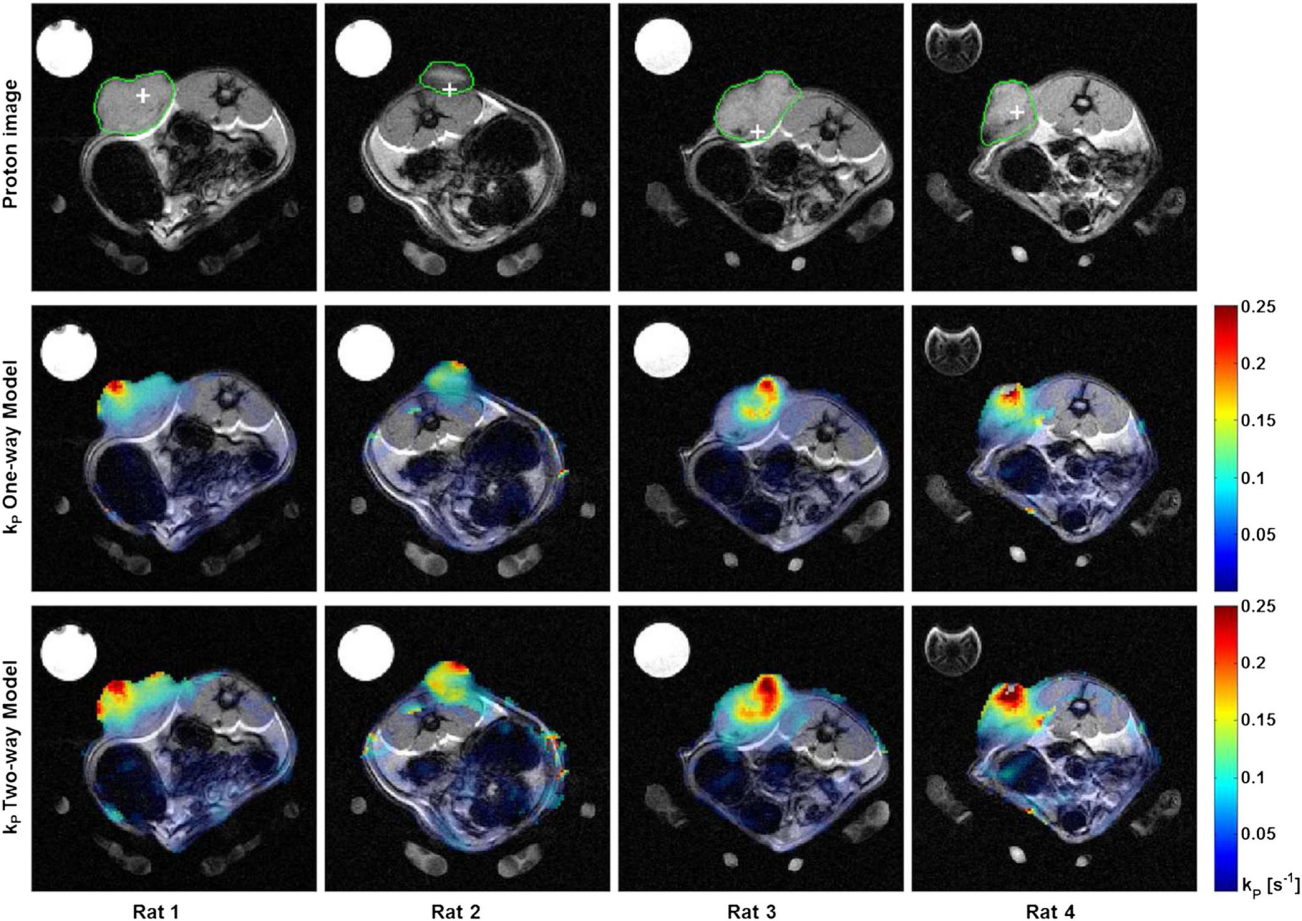
Functional parameter mapping in four rats with subcutaneous mammary adenocarcinomas demonstrating intratumoral heterogeneity. (a) Grey scale anatomical proton images showing the outline of the implanted tumours defining the ROI (green) and POI (white cross) used for modelling. (b) False‐colour functional maps of *k*_P_ calculated using the one‐way integral model superimposed over the grey scale anatomical imaging. (c) Similar colour maps using a two‐way integral model. The maximum *k*_P_ has been limited to 0.25 s^−1^ in both cases.

In the absence of a gold standard as a comparator, it was difficult to assess the model‐free parameters *in vivo*. However the differential model with a Heaviside step PIF was used as a surrogate standard, given its low AICc. Based on comparison with these calculated *k*_P_ values, the TTP significantly out‐performed the other approaches and the L–P ratio performed least well, being the only method to produce no significant correlation coefficients. The AUC also correlated very well with *k*_P_, with both correlation coefficients having *p* < 0.005. Parameter maps for *k*_P_ were generated for each of the four rats and are shown in Figure 4: *in vivo* parameter mappings derived from the model‐based methods were largely similar, and two representative examples are shown using the one‐way and two‐way non‐PIF integral models. Unfortunately, many of the model‐free parameter mapping methods were very sensitive to noise in regions of low lactate, rendering them difficult to interpret, which is an inherent limitation of some of these more simplified approaches. A visual inspection of the two sets shows that both models were able to distinguish the implanted tumours from normal tissue; however, the two‐way maps appeared to better highlight the full tumour and offered higher contrast‐to‐noise ratio, showing increased sensitivity over the one‐way model. *k*_P_ values were capped at 0.25 s^−1^, a value chosen to be suitably above those previously reported in rat tumours (~0.1 s^−1^) 28; only one rat (Rat 4) had an area of very high activity greater than this. The mean tumour *k*_P_ and range were both higher with the two‐way than the one‐way model, and in general the *k*_P_ maps demonstrated significant intra‐tumour heterogeneity.

## Discussion

Metabolic imaging with hyperpolarized ^13^C‐labelled molecules is an emerging clinical tool to non‐invasively detect real‐time metabolism. In order to utilize this dynamic data – either as a research technique or ultimately as a clinical tool – mathematical analysis methods are required that are sensitive to the small changes in metabolism that occur during tumour growth and following treatment, whilst being insensitive to noise and artefacts. Many of the approaches used to analyse the metabolism of hyperpolarized pyruvate are analogous to the methods used in other areas of MRI research (such as DCE‐MRI), as well as those used with PET. We have performed a comprehensive analysis of the main quantitative techniques used to analyse hyperpolarized data, as well as some novel approaches, with the aim of determining which are the most appropriate as research tools and which may have potential for clinical application in the future. Here we have used imaging data, rather than spectroscopic data, as this will be more applicable to patient studies, and have compared two methods of extracting the dynamic time‐course data from these images.

The approaches we studied can be divided into two groups: model‐based and simpler model‐free analyses. Of the four kinetic model variants, the differential and integral two‐way models performed best, showing strong correlation with *in vitro* LDH enzyme concentration and producing the most accurate modelling *in vivo* (as determined by a low AICc). Furthermore, the *T*_1_ values produced by these models, which we expect to be constant *in vitro*, had the smallest ranges and SDs of those tested, implying good parameterization. The one‐way model is a very popular approach because it allows for the use of Michaelis–Menten kinetics in order to solve for real, as opposed to apparent, reaction rate constants. However, this requires an assumption that is not fulfilled by the hyperpolarized exchange reaction: that the reaction has reached chemical equilibrium 40. The one‐way model was found to be unstable at higher enzyme concentrations within the physiological range, producing divergent *k*_P_ values almost three times higher than those given by the two‐way models (Fig. 2c). This divergence was also seen in some of the *in vivo* data sets (Fig. 4, Rat 4), and is likely to be a consequence of the approximation *k*_L_ = 0, which becomes less valid with increasing enzyme activity or in the presence of large pools of lactate that are freely exchanging with the pyruvate. A previous comparative study by Harrison *et al.* 41 demonstrated the shortcomings of the one‐way model by showing that results from fitting to hyperpolarized data were incompatible with those from mass spectrometry data. This suggests an inconsistency between the model and the underlying biology; the breakdown of the model at high enzyme activity that we have observed here provides further evidence against the use of the one‐way model.

We also examined the effect of explicitly modelling the pyruvate inflow within both the differential and integral (piecewise) models. Kazan *et al.* suggest modelling the PIF with a gamma‐variate function, which, although an excellent descriptor for the flow profile, requires accurate measurement of the arterial input function either with an invasive arterial line, or by image acquisition from a large vessel, which may be difficult to obtain from human hyperpolarized data 36. We therefore looked at incorporating simpler functions. From calculations of the AICc, the differential model with a Heaviside step PIF was significantly more likely to correctly describe the data than either variant of the piecewise model. The fitted pyruvate inflow end‐time *t*_e_ in this model correlated poorly with the measured injection length; this mismatch is indicative of the difficulty in fitting theoretical arterial input functions to hyperpolarized data with a low SNR and temporal resolution. Complicated inflow functions introduce several additional free parameters, which overfit noisy data and increase the error of the derived rate constants. With the current resolution limitations for clinical hyperpolarized imaging, simple approximations for pyruvate inflow, such as those used in this study, are therefore required.

The above methodologies are desirable when accurate fitting is required for research purposes; however, kinetic modelling can be computationally intensive and time consuming. We tested four model‐free methods as candidates for providing simple, robust parameters that are fully representative of the metabolic exchange reaction. Such approaches may allow easy intra‐patient and inter‐patient comparison across clinical sites. The *FmR*_*α*_ method described by Pagès and Kuchel 23 provided a better correlation with LDH concentration and tighter constraints on *T*_1_ than two of the modelling methods. However, in our study it was necessary to calculate *α* explicitly in each case, rather than using a constant value as suggested by the authors, to produce realistic results. Simpler still, and producing similar statistical results, is the AUC ratio described by Hill *et al.* 24, which is independent of the PIF. The lactate‐to‐pyruvate ratio at the time of maximum lactate signal was consistently the poorest method for describing the data. The final approach using TTP showed the strongest correlation with enzyme concentration *in vitro* of any analysis and the strongest correlation with *k*_P_ values derived using the differential PIF model *in vivo* of any model‐free method. The small size of the *in vivo* dataset means it is not possible to claim significance of the TTP over the AUC, and so further testing of these two methods on larger *in vivo* data sets is required. Nonetheless, these preliminary results along with the *in vitro* results suggest that the TTP may prove to be a very robust quantitative marker that is able to provide an equivalent assessment of metabolism to fitting a full PIF kinetic model.

Finally, we looked at two methods for extracting dynamic time‐course data from the images and assessed them for their effect on quantification. Data extracted using the ROI method provided universally stronger correlations with the *in vitro* enzyme concentrations than the POI method, although this was not significant in the majority of cases. Averaging results over a tumour ROI may provide a more sensitive measure of metabolic change in that region which is robust to experimental noise; however, it tells nothing of the local heterogeneity nor does it allow for tumours outside the region to be detected in the way that pixel‐by‐pixel analysis could. Furthermore, determining the anatomical limits of a tumour is subjective, and therefore difficult to reproduce accurately, even if performed by the same operator using the same dataset 42, 43. Our findings suggest that extracting data from a single POI is sufficiently robust to provide a metric that reports on the whole area, and may provide a simple and reproducible method for analysing the data objectively over time. This approach is analogous to the SUV_max_ used routinely in the analysis of clinical PET data. Under normal conditions, the POI (representing the highest lactate concentration over time) will lie within the boundaries of the tumour. Although unlikely, theoretically the POI could lie outside the tumour margin if it was so poorly vascularized that the time required to perfuse the tumour was long compared with the half‐life of the polarized pyruvate signal, or if the systemic lactate level was very markedly elevated. Since single‐pixel analysis was shown to be sufficiently robust, parameter mapping of *k*_P_ was performed in the rats. This showed marked intratumoral heterogeneity, with some areas of low exchange within the tumours and regions of high activity extending beyond the tumour boundary. Due to the spatial resolution of the ^13^C images, it is not possible to determine whether these findings are due to artefacts from partial voluming or whether there is underlying biological heterogeneity due to changes in metabolism or vascularity, although a highly heterogeneous vasculature is a known feature of the subcutaneous tumour type studied here 44.

The rate constant *k*_P_ is generally considered to be the gold standard for quantification of hyperpolarized data, but several of the simpler non‐model‐based approaches used here appeared to provide a good approximation to the reaction. A good probe for tumour biology should be repeatable, reproducible, quantitative, highly sensitive and highly specific. The results of this study show that *k*_P_ is a robust biomarker of LDH activity. However, at low LDH concentrations the modelling is more susceptible to noise, and at high LDH concentrations greater divergence was demonstrated, which may relate to undersampling. Importantly, this work has shown that these modelling methods performed well in the physiological range, and further research is required from human studies to validate this. Variation in *k*_P_ may occur secondary to other factors such as SNR, which is dependent on polarization 45, 46, pyruvate concentration 35 and imaging protocol 47. Therefore the optimization and standardization of each of these is an important step for multi‐centre comparison, or if *k*_P_ is to be considered as a clinical tool.

In conclusion, for accurate kinetic analysis of the hyperpolarized pyruvate–lactate exchange reaction *in vivo*, the two‐way differential model with a Heaviside step PIF centred on the pyruvate peak was best able to characterize the data with the fewest free parameters. If the data prior to the pyruvate peak is not included in the analysis, the two‐way differential and integral models performed equally well and were significantly better than the one‐way model in the presence of high enzyme activity or when applied to the pixel‐by‐pixel analysis. As a simple parameter for clinical quantification of hyperpolarized imaging data, the TTP performed best both *in vitro* and *in vivo*, providing excellent correlation with model‐derived *k*_P_ values. Extracting data from an averaged ROI may provide the most sensitivity to small changes in metabolism; however, the POI approach is sufficiently robust to be applied pixel‐by‐pixel, allowing tumour heterogeneity to be probed. This work provides a basis for analysing data from future human trials in hyperpolarized ^13^C imaging.
